# Electrocardiography clues in assessment of patients with premature ventricular contractions

**DOI:** 10.3906/sag-2012-70

**Published:** 2021-09-07

**Authors:** Ahmet AKDİ, Bahar TEKİN TAK, Elif Hande ÖZCAN ÇETİN, Mehmet Serkan ÇETİN, Çağrı YAYLA

**Affiliations:** 1 Department of Cardiology, Ankara City Hospital, Ankara, Turkey; 2 Department of Cardiology, Etimesgut State Hospital, Ankara, Turkey; 3 Department of Cardiology, Ankara City Hospital, University of Health Sciences, Ankara, Turkey

**Keywords:** Fragmented QRS, heart rate variability, Holter monitoring, premature ventricular contraction, Tp-e /QT ratio

## Abstract

**Background/aim:**

Some electrocardiography (ECG) parameters such as Tp-e interval, Tp-e / QT ratio, fragmented QRS (fQRS), and heart rate variability (HRV) are related to cardiovascular mortality and morbidity. We aim to investigate the relation between premature ventricular contraction burden and these parameters on 24-h ECG recording.

**Materials and methods:**

The study is a retrospective investigation of the 24-h Holter ECG and echocardiography of 199 patients who underwent the procedures due to complaints of palpitation. A frequency of < 10% PVCs / 24 h was classified as seldom group (98 patients), while > 10% PVCs / 24 h was designated as frequent group (101 patients).

**Results:**

Tp-e interval was significantly longer (62 [54–78] vs 75 [60–84], p < 0.001), Tp-e / QT ratio was significantly increased (0.18 [0.16–0.20] vs 0.21 [0.18–0.22], p = 0.001) in frequent PVC group. The percentage of fQRS was significantly increased in frequent PVC group (30.6% vs 47.5%, p = 0.015). When the groups were compared, no significant difference was found in HRV time domain indices. Positive correlations were observed between PVC burden and Tp-e (r = 0.304, p < 0.001), Tp-e / QT (r = 0.275, p < 0.001).

**Conclusion:**

Our study showed that Tp-e interval, Tp-e / QT and fQRS are associated with frequency of PVCs. These measurements in patients with PVCs may form part of assessment of cardiovascular risk.

## 1. Introduction

Premature ventricular contractions (PVCs) are commonly seen in the electrocardiography (ECG) of patients who are asymptomatic or have symptoms such as palpitation, dizziness, dyspnea, and chest pain. They occur in about 4% of the general population in various studies [1]. It is shown that frequent PVCs have a good prognosis in the absence of structural heart disease [1], but according to some studies, frequent idiopathic PVCs can cause an increased risk of mortality in patients with or without structural heart disease [2,3]. In recent studies, patients with frequent PVCs have reported left ventricular (LV) dysfunction [4], and malignant ventricular arrhythmias in patients with structural heart disease [5].

It has been reported that several parameters that can be provided on ECG are related to cardiovascular mortality and morbidity. Myocardial repolarization markers, such as Tp-e interval and Tp-e/QT ratios [6,7], fragmented QRS (fQRS) [8] and heart rate variability (HRV) [9] are https://drive.google.com/drive/folders/0Bz7lygGrpTxrVWMzSjFZTnczZmcassociated with increased cardiovascular morbidity and mortality. These simple, easily accessible, inexpensive, and noninvasive parameters may be useful indexes of ventricular arrhythmia in patients with PVCs that is caused LV dysfunction. In this study, we investigated the relation between PVC burden and these ECG markers that simultaneously can be provided from 24-h ECG recording.

## 2. Methods

### 2.1. Study population

We evaluated retrospectively 199 patients who underwent 24-h ambulatory Holter monitoring because of complaints of palpitation in our hospital. Burden of PVC was calculated as the total number of PVCs divided by the number of all QRS complexes in the total recording time. A frequency of < 10% PVCs/24 h was designated as seldom (control) group (n = 98), >10% PVCs/24 h was designated as frequent group (n = 101). In the frequent group, 51 (50.5%) of patients were using antiarrhythmic drugs, 39 of which were b-blocker; however, none of patients were using antiarrhythmic drugs in the control group. Patients who have either an atrial fibrillation/flutter, bundle branch block, permanent pacemaker therapy, acute coronary syndrome, severe valvular heart disease, cardiomyopathy, congenital heart disease, pulmonary hypertension, thyroid disorder, malignancy, or electrolyte disturbance were excluded. The study complied with the principles outlined in the Declaration of Helsinki and the local ethics committee.

### 2.2. Holter recordings and electrocardiographic analysis

Holter recordings were performed by using a three-channel digital recorder (Del Mar Reynolds Medical Ltd, Hertford, UK). All patients were asked to do their normal daily activities and normal sleep rhythm during Holter monitoring and warned not to smoke, not to consume alcohol or coffee during the Holter recording. After computerized primary analysis, the recordings were edited manually, evaluated for PVCs, and the number of PVCs was recorded. It was validated that the lowest PVC burden resulting in a reversible cardiomyopathy was 10% previously [10]; therefore, frequent PVCs were defined as PVC burden greater than 10%. The records lasting for least 22 h and of sufficient quality for evaluation were included in the analysis. If these criteria were unsuccessful, the Holter monitoring was repeated. We measured the following time domain indices of HRV in the 24-h recordings: the standard deviation of all normal-to-normal RR intervals (SDNN, ms), the standard deviation of 5-min mean RR intervals (SDANN, ms), percentage of successive normal RR intervals exceeding 50 ms (pNN50, %), and the square root of the mean of the squares of the differences between successive normal-to-normal RR intervals (RMSSD, ms).

Twelve-lead ECGs were acquired at 10 mm/mV amplitude and 25 mm/s (Hewlett Packard, Page-writer, CA, USA) rate, with the patient in the supine position. All ECGs were transferred to a personal computer through a scanner and then used for × 300% magnification using Adobe Photoshop software. The recordings were analyzed by two cardiologists who were blinded to echocardiographic and clinical data of the study patients. We excluded subjects with U waves in their ECGs from the study and calculated the average value of least two readings for each lead. QT interval was defined as the time from the start of the QRS wave to the end of the T wave to the point at which the T wave returned to the isoelectric line and corrected QT interval was calculated by using Bazett’s formula (cQT = QT / √R - R interval). The tail method was used in the study because the tail method is a better predictor of mortality than the tangent method in the measurement of Tp-e interval [11]. Tp-e interval was defined as the interval from the peak to the end of the T wave to the point where the wave returned the isoelectric line [12]. The measurement of the Tp-e interval was obtained from leads V2 and V5. Corrected Tp-e interval was also calculated by using Bazett’s formula (cTp-e = Tp-e interval / √RR interval). The Tp-e/QT and Tp-e/cQT ratios were calculated from these measurements. fQRS was described as an additional R wave (R’) or notching of the R wave or S wave, or over one R’ without typical bundle branch block in two contiguous leads suitable to a major coronary artery area [8].

### 2.3. Echocardiography

We performed all echocardiography examinations with a Vivid 7 Dimension Cardiovascular Ultrasound System (General Electric Vingmed, Horten, Norway) in our institution according to guidelines of the American Society of Echocardiography [13]. Left ventricular end-diastolic diameter, left ventricular end-systolic diameter, interventricular septum and posterior wall thickness in diastole, ejection fraction (EF), and left atrium diameter in apical for chamber dimensions were evaluated and noted.

### 2.4. Statistical analysis

All analyzes were performed by using SPSS software program (version 26.0, SPSS, Chicago, İllinois, USA). Categorical variables were expressed as numbers and percentage. All continuous variables were tested with the Kolmogorov–Smirnov test to determine the distribution. Nonparametric variables were expressed as median and interquartile range (IQR), while parametric variables were expressed as means ± standard deviation. Categorical variables were compared among groups using the chi square (χ^2^) test. Continuous variables among groups were compared using the independent-samples student’s t-test when the distribution was normal, Mann–Whitney U test when the distribution was skewed. Pearson correlation analysis was performed to analyze the relationship between Holter parameters and PVC burden. A multivariate logistic regression analysis, which included variables with p < 0.1 in univariate analysis, was performed to determine independent predictors of frequent PVCs. Statistical significance was accepted as a p value of < 0.05.

## 3. Results

The study consisted of 199 patients with a mean age of 49.14 ± 14.53 years and was divided into two groups as seldom and frequent PVC burden. The groups contained 98 (62.2% female; mean age 48.09 ± 13.13 years) and 101 (55.4% female; mean age 50.15 ± 15.76 years) patients, respectively. The baseline clinical, laboratory and echocardiographic characteristics of the two groups are shown in Table 1. Only left ventricular EF was significantly lower in Frequent PVC group (58.78 ± 6.40 vs 56.75 ± 6.41, p = 0.027), but no significant difference was found among the two groups in terms of another baseline clinical and laboratory characteristic.

Ambulatory ECG parameters among the groups are summarized in Table 2. In the comparison of ECG parameters among the groups, while the QT interval was no significant difference (p = 0.268), cQT interval was significantly longer in frequent PVC group than in seldom PVC group (p = 0.025). Tp-e interval was significantly longer in frequent PVC group (62 [54–78] vs 75 [60–84], p < 0.001) and both Tp-e/QT (0.18 [0.16–0.21] vs 0.21 [0.18–0.22], p = 0.001) and Tp-e/cQT (p = 0.016) ratio were significantly increased in frequent PVC group. The percentage of patients with fQRS was significantly increased in frequent PVC group (30.6% vs 47.5%, p = 0.015). When the groups were compared, no significant difference was found in HRV time domain indices (SDNN, SDANN, RMSSD, pNN50).

Correlation analysis between PVCs burden and electrocardiographic parameters are shown in Table 3. According to Pearson’s correlation test, positive correlations were observed between PVC burden and Tp-e (r = 0.304, p < 0.001), Tp-e/QT (r = 0.275, p < 0.001) and Tp-e/cQT (r = 0.223, p < 0.001) (Figure). Any HRV time domain indices were not correlated with PVCs burden. In the multivariate logistic regression analysis, Tp-e/QT ratio (OR: 1.11, 95%CI: 1.03–1.21, p = 0.007), ejection fraction (OR: 0.94, 95%CI: 0.89–0.99, p = 0.009) and fQRS (OR: 1.92, 95%CI: 1.02–3.60, p = 0.041) were found as predictors of frequent PVCs (Table 4).

## 4. Discussion

The main findings of the present study were that Tp-e interval, Tp-e/QT ratios, and presence of fQRS were significantly higher in patients with frequent PVC burden group, though HRV time domain indices, which reflect vagal modulation, were not different between the groups. Both prolongation in the dispersion of myocardial repolarization and fQRS predispose the malignant ventricular arrhythmia and have prognostic importance in mortality. They are associated with cardiac mortality and morbidity in various cardiac conditions [8,14].

PVCs are frequent causes of palpitations and observed on 40%–75% of Holter monitors and 1% of 12-lead ECGs in healthy individuals [15]. The frequency of PVCs increases with age; the prevalence among 45–65 years is approximately 6% [9]. Various factors which disrupt the stability of the myocardium, such as altered hemodynamic status, increased sympathetic tonus, or electrolyte imbalances, may cause PVCs to transform into ventricular arrhythmia [16]. That Savelieva et al. [17] found significant QT turbulence after PVC in patients with a structurally healthy heart, may provide information on the cause of malignant arrhythmias in these patients. PVCs are strongly associated with ventricular dilatation and dysfunction [18]. Dilated and tensed left ventricle is thought to be an important role in the development of arrhythmias [19]. LV dysfunction can cause increased automaticity, reentry, or triggered activity, which plays a major role in the occurrence of arrhythmias.

Primary finding of this study was the relationship between myocardial repolarization markers and the frequency of PVCs. We found an increase in Tp-e interval, Tp-e/QT and Tp-e/cQT ratios as PVC frequency increased over 10% on a 24-h Holter recording. Karaman et al. [20] showed that Tp-e interval and Tp-e/cQT ratio increased in patients with high PVC number, but there was no significant difference in Tp-e/QT and EF between the groups. In our study, however, a significant difference has been found in all of them, Tp-e, Tp-e/cQT, Tp-e/QT, and EF between the groups. Furthermore, Yayla et al. [21] found that Tp-e interval, Tp-e/QT ratio, and Tp-e/cQT ratio significantly decreased after the successful RFA in patients with a PVC burden of over 5% on a 24 h Holter recording. Tp-e interval corresponds to the repolarizations of the epicardial and mid-myocardial cells. Therefore, Tp-e interval and Tp-e/QT ratio are a measurement that reflects the dispersion of repolarization. This mechanism may play a role in the patients with increased PVC frequency. This suggestion has also been supported by previous studies related to pathophysiological conditions accompanied by the heterogeneity of repolarization such as hypertrophic cardiomyopathy, Brugada syndrome, heart transplantation and long QT syndrome [22–24]. Prolongation of QT and cQT durations may be associated with polymorphic ventricular tachycardia, Torsades de pointes, and SCD [23,26]. The higher detection of Tp-e interval and Tp-e/QT ratio in patients with increased PVC frequency suggests that the risk of malignant arrhythmias might be higher in these patients.

It is shown that the Tp-e interval such as the QT interval is heart rate (HR) dependent [27]. Also, correcting the Tp-e interval using the Bazett’s formula (cTp-e = Tp-e interval / √RR interval) has improved the predictive value of this marker for SCA risk [28]. Mathematically, if ‘corrected Tp-e/corrected QT’ ratio is simplified by the common factor (√ RR interval) of the numerator and denominator, Tp-e/QT ratio is obtained. Thus, the Tp-e/QT ratio remains constant despite dynamic physiological changes in HR, and it may be a ventricular repolarization index that is independent of HR.

Other finding of the present study was that fQRS on ECG was related to frequent PVCs. In a previous study, Temiz et al. found that fQRS and reduced EF were independently associated with frequent PVCs [29], our study also supports these findings. fQRS was found to be associated with ventricular arrhythmias in patients with various cardiac disorders, such as chronic heart failure, hypertrophic cardiomyopathy, Brugada syndrome, and idiopathic ventricular fibrillation [30–32]. Myocardial fibrosis and/or ischemia which the altered homogeneity of myocardial electrical activity is accepted as being responsible for fQRS formation [33]. That fQRS increase PVC frequency, which are associated with left ventricular dilatation and dysfunction [18], supports this mechanism. We also found that patients with frequent PVCs have lower EF values and higher.

Another finding of the present study was related to HRV in 24-h ECG records. We evaluated HRV to the frequency of PVCs, but no differences were observed between the groups in terms of HRV time domain indices reflecting vagal modulation. In a previous study, in parallel with our findings, Askın et al. did not observe differences between occasional and frequent PVCs in terms of HRV time domain indices [34].

This study has some limitations. Primarily, this was a retrospective study with a relatively small population. Since we could not follow up on the patients for future arrhythmic events, we did not have data on cardiac events for this study. Primarily, this was a retrospective study with a relatively small population. Since we could not follow up on the patients for future arrhythmic events, we did not have data on cardiac events for this study. Otherwise, while none of the patients in the seldom group were using antiarrhythmic drugs, nearly half of the patients in the frequent group were using an antiarrhythmic drug, which could affect the outcome. Further comprehensive and prospective follow-up studies are needed to make clear the clinical importance of these factors in patients with PVCs. Then, time domain indices for HRV were evaluated, but frequency domain indices were not evaluated in the study. With both time and frequency domain indices, HRV can be analyzed more comprehensive.

In conclusion, our study showed that myocardial repolarization markers (Tp-e interval, Tp-e/QT) and fQRS are associated with frequent of PVCs. These measurements, which show that myocardial electrical activity is impaired, in patients with PVCs may form part of a comprehensive approach for the assessment of cardiovascular risk. So, it needs further comprehensive studies to make clear the clinical importance of these factors.

## Figures and Tables

**Figure:**
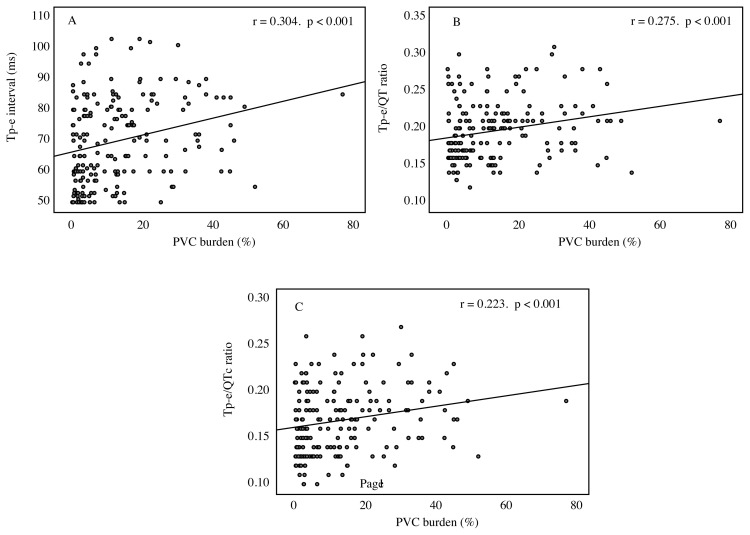
Correlation between Tp-e interval (A). Tp-e / QT ratio (B). Tp-e / cQT ratio (C) and premature ventricular complex burden. cQT: corrected QT; PVC: premature ventricular contraction. Tp-e: T wave peak-to-end interval.

**Table 1:** Baseline clinical and laboratory parameters of the study population.

	Seldom PVC (n = 98)	Frequent PVC (n = 101)	p value
Age, years	48.09 ± 13.13	50.15 ± 15.76	0.319
Sex, female n (%)	61(62.2)	56 (55.4)	0.330
Hypertension, n (%)	37(37.8)	47 (46.5)	0.210
Diabetes mellitus, n (%)	21(21.4)	30 (29.7)	0.181
CAD, n (%)	24(24.5)	35 (34.7)	0.117
Smoking, n (%)	23(23.5)	25 (24.8)	0.595
Heart rate, bpm	80.93 ± 14.65	83.04 ± 14.34	0.305
LVEF, %	58.78 ± 6.40	56.75 ± 6.41	0.027
Hemoglobin, g/dL	14.00(13.00–14.50)	14.10 (13.00–14.60)	0.497
Leukocyte, ×103 /mL	8.12 ± 4.74	7.62 ± 1.82	0.319
Glucose, mg/dl	95.41 ± 19.54	97.25 ± 16.31	0.474
Creatinine, mg/dL	0.95 ± 0.33	0.94 ± 0.25	0.824
Sodium, mmol/L	139.83 ± 2.81	140.37 ± 2.44	0.150
Potassium, mmol/L	4.66 ± 3.73	4.34 ± 0.40	0.401
LDL-C, mg/dL	113.97 ± 40.71	109.48 ± 37.45	0.420
HDL-C, mg/dL	51.71 ± 15.56	50.90 ± 10.19	0.663
Triglyceride, mg/dL	145.39 ± 75.28	120.0(102.0–168.5)	0.396
TSH, UI/mL	2.07 ± 2.65	1.79 ± 1.11	0.341

CAD: Coronary artery disease; HDL-C: high-density lipoprotein cholesterol; LFEF: left ventricle ejection fraction; LDL-C: low-density lipoprotein cholesterol; PVC: premature ventricular contraction; TSH: thyroid-stimulating hormone.

**Table 2:** Parameters of ambulatory electrocardiography monitoring of the study population.

	Seldom PVC (n = 98)	Frequent PVC (n = 101)	p value
PVC burden, %	3.767 ± 2.123	23.462 ± 12.450	<0.001
fQRS, n (%)	30(30.6)	48 (47.5)	0.015
QT, ms	350(330–380)	362(330–385)	0.268
cQT, ms	410(393–430)	420(400–450)	0.025
QTd, ms	25(20–32)	28(21–35)	0.037
Tp-e, ms	62(54–78)	75(60–84)	<0.001
cTp-e, ms	72(6–86)	86(73–99)	<0.001
Tp-e/QT	0.18(0.16–0.21)	0.21(0.18–0.22)	0.001
Tp-e/cQT	0.16(0.13–0.19)	0.17(0.15–0.20)	0.016
HRV time domain indices			
SDNN, ms	126.133 ± 32.121	123.53 ± 33.924	0.580
SDANN, ms	60.5(42.0–106.0)	68(53–122)	0.067
RMSSD, ms	27.0(20.0–36.0)	31(22–42)	0.089
pNN50, %	7(2–13)	9(3–17)	0.152

cQT: corrected QT; cTp-e: corrected Tp-e; fQRS: fragmented QRS; HRV: heart rate variability; PVC: premature ventricular contraction; QTd: QT dispersion; Tp-e: T wave peak-to-end interval.

**Table 3:** Correlation between PVC burden and electrocardiographic parameters.

Variables	Spearman’s correlation coefficient	p Value
Age	0.078	0.272
QT	0.097	0.175
cQT	0.152	0.032
QTd	0.239	0.001
Tp-e	0.304	< 0.001
cTp-e	0.298	< 0.001
Tp-e/QT	0.275	< 0.001
Tp-e/cQT	0.223	< 0.001
HRV time domain indices		
SDNN	−0.046	0.552
SDANN	0.086	0.228
PNN50	0.059	0.404
rMSSD	0.077	0.281

cQT: corrected QT; cTp-e: corrected Tp-e; PVCs: premature ventricular contractions; HRV: heart rate variability; QTd: QT dispersion; Tp-e: T wave peak-to-end interval.

**Table 4:** Univariate and multivariate analyses for predictors of frequent PVC.

Variables	Univariate analysis		Multivariate analysis	
	OR (95% CI)	p value	OR (95% CI)	p value
Age	1.01(0.99–1.03)	0.38		
Sex, male	1.32(0.75–2.33)	0.330		
Hypertension	0.70(0.40–1.22)	0.211		
Ejection fraction	0.95(0.91–1.00)	0.031	0.94(0.89–0.99)	0.009
CAD	0.61(0.33–1.13)	0.118		
fQRS	2.05(1.15–3.67)	0.015	1.92 (1.02–3.60)	0.041
Tp-e/QT ratio	1.13(1.05–1.21)	0.002	1.11(1.03–1.21)	0.007
HRV (SDNN)	0.99(0.99–1.01)	0.578		

CAD: Coronary artery disease; CI = confidence interval; fQRS: fragmented QRS; OR: odds ratio; PVC: premature ventricular contraction. HRV: heart rate variability; Tp-e: T wave peak-to-end interval.
